# Factors influencing receipt and time to treatment of immunotherapy relative to chemotherapy in stage III and stage IV melanoma

**DOI:** 10.1002/cam4.6888

**Published:** 2024-01-08

**Authors:** Gurman S. Dhaliwal, Ahmad B. Shahin, Elisabeth S. Lim, Lanyu Mi, Aaron R. Mangold, David L. Swanson, Collin M. Costello

**Affiliations:** ^1^ Department of Dermatology Mayo Clinic Scottsdale Arizona USA; ^2^ Mayo Clinic Alix School of Medicine Scottsdale Arizona USA; ^3^ Department of Qualitative Health Science Scottsdale Arizona USA

**Keywords:** chemotherapy, health disparity, minority and vulnerable populations, immunotherapy, melanoma, minority health, rural population

## Abstract

**Background:**

Immunotherapies have changed the landscape of late‐stage melanoma; however, data evaluating timely access to immunotherapy are lacking.

**Methods:**

A retrospective cohort study utilizing the National Cancer Database was conducted. Stage III and IV melanoma cases diagnosed between 2011 and 2018 that received systemic treatment with either immunotherapy or chemotherapy were included. Chemotherapy included BRAF/MEK inhibitors. Multivariable logistic regression models were utilized to evaluate factors associated with the likelihood of receiving immunotherapy as primary systemic treatment relative to chemotherapy; additionally, Cox proportional hazards models were utilized to incorporate time from diagnosis to primary systemic therapy into the analysis.

**Results:**

The study population was comprised of 14,446 cases. The cohort included 12,053 (83.4%) immunotherapy and 2393 (16.6%) chemotherapy cases. In multivariable logistic regression analysis, factors significantly associated with immunotherapy receipt included population density, circle distance, year of diagnosis, Breslow thickness, and cancer stage. Immunotherapy timing was evaluated using multivariable Cox regression analysis. Minorities were less likely to receive timely immunotherapy than non‐Hispanic Whites (HR 0.83, CI 0.74–0.93, *p* = 0.001). Patients at circle distances of 10–49 miles (HR 0.94, CI 0.89–0.99, *p* = 0.02) and ≥50 miles (HR 0.83, CI 0.77–0.90, *p* < 0.001) were less likely to receive timely immunotherapy.

**Conclusion:**

Patients traveling ≥10 miles and minorities have a decreased likelihood of receiving timely immunotherapy administration for primary systemic treatment. Future research is needed to identify what barriers and approaches can be leveraged to address these inequities.

## 1 INTRODUCTION

Advances in immunotherapies have dramatically changed the therapeutic landscape of melanoma.^1^ Despite these advances, vulnerable populations have experienced increased morbidity and mortality, partly due to melanoma diagnosis and treatment delays.^2^ Additionally, they are less likely to receive immunotherapy.^3^

Few reports have evaluated sociodemographic factors associated with immunotherapy, with each study sourcing data from the National Cancer Database (NCDB).^3^, ^4^, ^5^, ^6^, ^7^ However, the majority included periods of time before modern immunotherapies. The current knowledge gaps within the literature pertaining to immunotherapy utilization include factors associated with immunotherapy utilization as primary treatment relative to targeted chemotherapy, factors associated with time to immunotherapy utilization, and an updated analysis confined to modern immunotherapy.

We conducted a study comparing the utilization of modern immunotherapy relative to targeted BRAF/MEK inhibitors/chemotherapy in stage III and IV melanoma cases and evaluated factors influencing the timing of immunotherapy administration.

## 2 METHODS

This study was exempt by Mayo Clinic's institutional review board, as it was a retrospective cohort study using de‐identified NCDB data. Inclusion criteria were stage III and IV melanoma cases diagnosed between 2011 and 2018 that involved treatment with either immunotherapy or chemotherapy, 2018 representing the most recent year data were available. Ipilimumab was approved by the Federal Drug Administration in 2011; in 2016, immunotherapy was recommended as first‐line therapy.^8^ Within the NCDB, chemotherapy includes targeted BRAF/MEK inhibitors. Exclusion criteria included cases not managed with immunotherapy or chemotherapy, missing racial/ethnic data, and lacking insurance (Figure S1). Uninsured patients were excluded as they comprised 3% of the study population. Including them in the statistical analysis could have led to bias due to the small sample size. Each case was classified as either “immunotherapy” or “chemotherapy” based on the primary systemic treatment modality; immunotherapy or chemotherapy was defined as either the only systemic treatment or the first systemic treatment prescribed for a particular patient. Based on race/ethnicity data, cases were classified as “non‐Hispanic White (NHW)” and “minority,” which included all other racial subgroups. Insurance status was subcategorized for each case as private or governmental, including Medicaid, Medicare, and “other governmental insurance.” The United States Department of Agriculture (USDA) has defined all counties in the United States as metropolitan, urban, and rural. The NCDB uses those predefined county labels. Metropolitan counties encompass a metro area, usually with a population fewer than 250,000 or more people. Urban counties are those with a population usually ranging from 2500 to 20,000, while rural counties are those with a population of less than 2500.

Patient demographics and clinical characteristics were summarized with median (IQR) for continuous variables and count (percent) for categorical variables. Circle distance was measured among the data collected; circle distance was defined as the distance in miles between the patient's zip code and the hospital that reported the case. Wilcoxon rank sum test or chi‐squared test were used for comparison as appropriate. Multivariable logistic regression models were utilized to explore factors associated with the likelihood of receiving immunotherapy as primary systemic therapy versus chemotherapy, as the reference group, for the cohort of patients diagnosed with stage III and stage IV melanoma between 2011 and 2018. Additionally, Cox proportional hazards models were utilized to incorporate time from diagnosis to primary systemic therapy into the analysis of factors associated with the utilization of immunotherapy as primary systemic therapy. Analyses were conducted using SAS 9.4 (SAS Institute, Inc., Cary, NC). All tests were two‐sided; we considered *p*‐values <0.05 as significant.

## 3 RESULTS

In total, the study population was comprised of 14,446 cases. The cohort had 12,053 (83.4%) immunotherapy cases and 2393 (16.6%) chemotherapy cases. Complete descriptive statistics of the study population are presented in Table S1.

**FIGURE 1:**
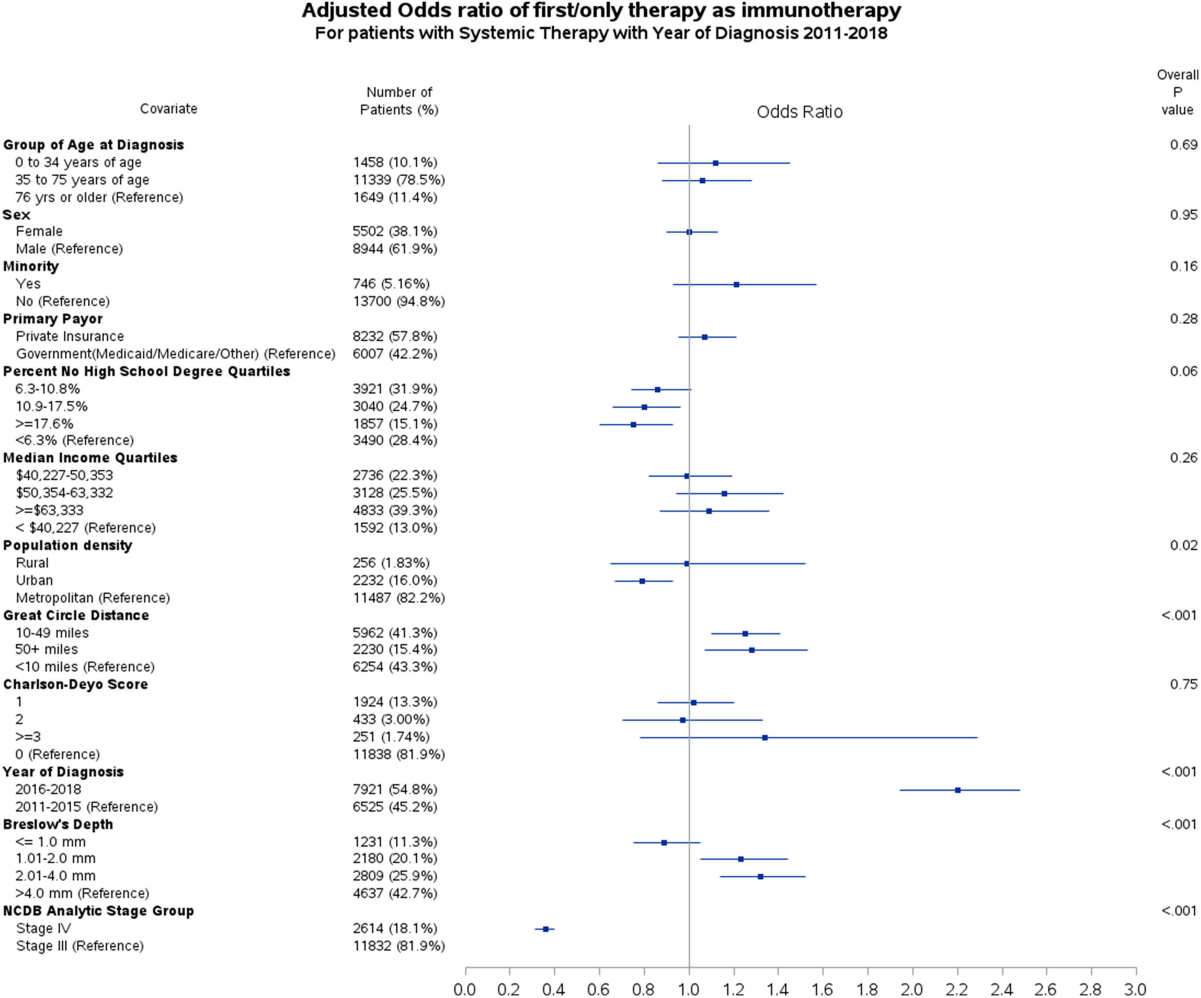
Adjusted odds ratio plot for patients who received immunotherapy compared to chemotherapy for stage III and IV melanoma between the years 2011 and 2018.

In multivariable logistic regression analysis (Figure 1), factors associated with the likelihood of receiving immunotherapy as primary systemic therapy included population density, circle distance, year of diagnosis, Breslow thickness, and cancer stage. Those residing within urban populations were less likely to receive immunotherapy (OR 0.79, CI 0.67–0.93, *p* = 0.01). Conversely, there was no difference in immunotherapy receipt between residents of rural and metropolitan populations (OR 0.99, CI 0.65–1.52, *p* = 0.98). Circle distances of 10–49 miles (OR 1.25, CI 1.10–1.41, *p* < 0.01) and ≥50 miles (OR 1.28, CI 1.07–1.53, *p* = 0.01) were associated with a higher likelihood of receiving immunotherapy. Patients managed between 2016 and 2018 were more likely to receive immunotherapy than those managed between 2011 and 2015 (OR 2.20, CI 1.94–2.48, *p* < 0.001). Patients with T2 (1.01–2.0 mm) and T3 (2.01–4.0 mm) tumors were more likely to receive immunotherapy (OR 1.23, CI 1.05–1.44, *p* = 0.01 and OR 1.32, CI 1.14–1.52, *p* < 0.001, respectively). Patients with stage IV tumors were less likely to receive immunotherapy than those with stage III tumors (OR 0.36, CI 0.31–0.40, *p* < 0.001). When comparing stage III and IV, stage IV patients were older and more likely male, had Medicare insurance, residence in areas with lower education levels and median income, were more likely to be seen at a community cancer program or comprehensive community cancer program, and had higher Charlson–Deyo scores (Table S2).

**FIGURE 2:**
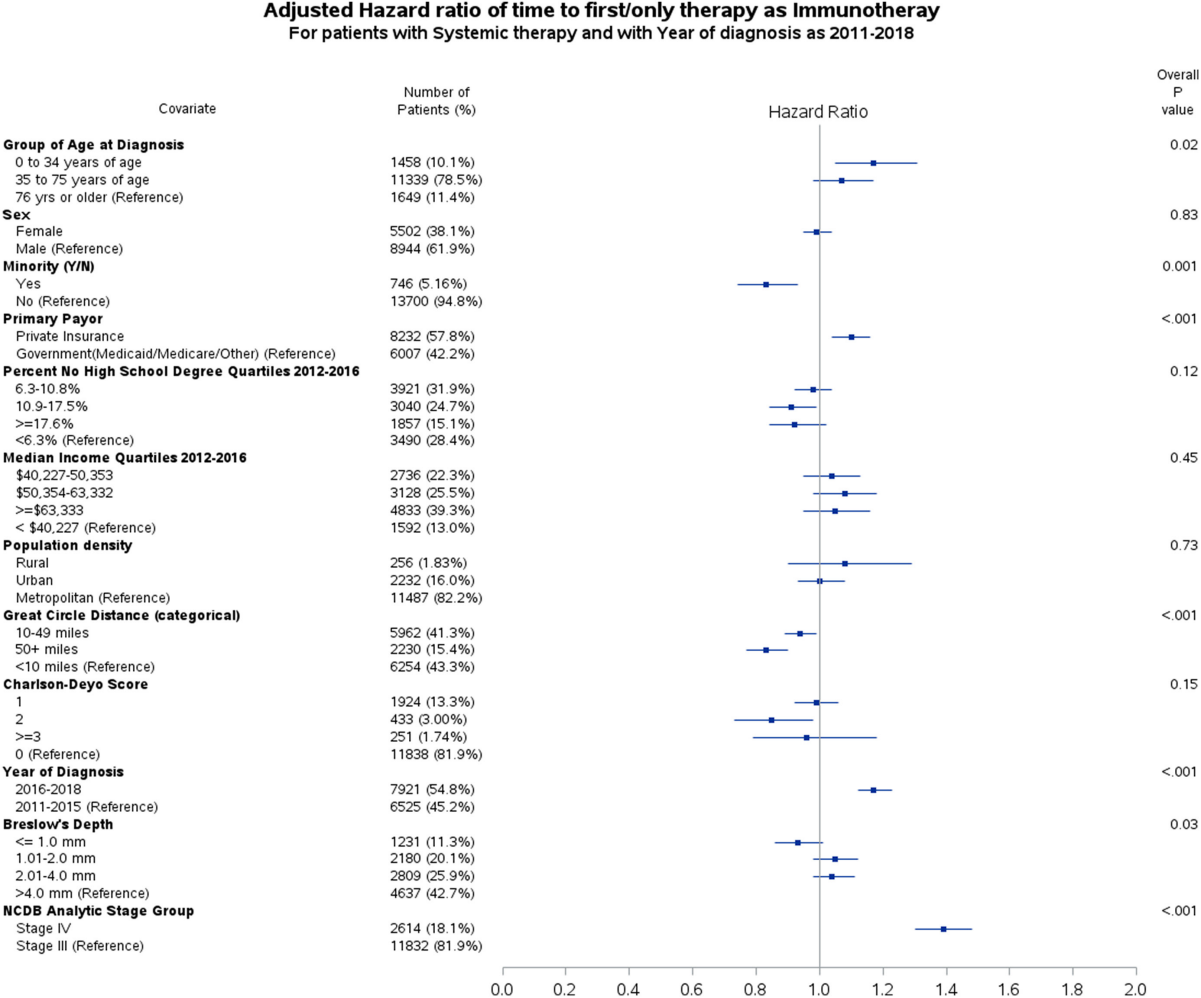
Adjusted hazard ratio plot for patients who received immunotherapy compared to chemotherapy for stage III and IV melanoma between the years 2011 and 2018.

To evaluate the time to immunotherapy, a multivariable Cox regression analysis was performed (Figure 2). The median time to immunotherapy from diagnosis was 3.55 months (CI 3.52–3.58). Age at diagnosis, race/ethnicity, insurance payor, circle distance, year of diagnosis, Breslow depth, and NCDB analytic stage were factors associated with the likelihood of timely receipt of immunotherapy. Patients aged <35 were more likely to receive timely immunotherapy than those aged >75 (HR 1.17, CI 1.05–1.31, *p* = 0.01). Minorities were less likely to receive timely immunotherapy (HR 0.83, CI 0.74–0.93, *p* = 0.001). Patients with private insurance were more likely to receive timely immunotherapy than those with governmental insurance (HR 1.10, CI 1.04–1.16, *p* = 0.001). Circle distances of 10–49 miles (HR 0.94, CI 0.89–0.99, *p* = 0.02) and ≥50 miles and (HR 0.83, CI 0.77–0.90, *p* < 0.001) were less likely to receive timely immunotherapy. Patients with stage IV melanoma were more likely to receive timely immunotherapy than those with stage III melanoma (HR 1.39, CI 1.30–1.48, *p* < 0.001). Lastly, Table 1 shows the time to immunotherapy for race/ethnicity, circle distance, stage, and primary payer.

**TABLE 1:** Time to immunotherapy.

Minorities and non‐Hispanic Whites		
	Months until immunotherapy (95% CI)^a^	*p*‐value^b^
Minorities	3 months: 32.4% (28.8%–35.8%)	<0.001
	6 months: 81.2% (77.7%–84.1%)	
	12 months: 98.2% (96.2%–99.1%)	
Non‐Hispanic Whites	3 months: 36.9% (36.0%–37.7%)	
	6 months: 88.7% (88.0%–89.3%)	
	12 months: 98.8% (98.5%–99.0%)	
Circle distance		
Less than 10 miles	3 months: 37.9% (36.6%–39.1%)	<0.001
	6 months: 88.9% (87.9%–89.8%)	
	12 months: 98.8% (98.3%–99.1%)	
10–49 miles	3 months: 36.5% (35.3%–37.8%)	
	6 months: 88.6% (87.6%–89.5%)	
	12 months: 98.9% (98.4%–99.2%)	
50 or more miles	3 months: 33.5% (31.4%–35.6%)	
	6 months: 85.9% (84.1%–87.4%)	
	12 months: 98.5% (97.6%–99.0%)	
Melanoma stage		
Stage IV	3 months: 51.9% (49.7–54.0%)	<0.001
	6 months: 87.9% (86.1–89.5%)	
	12 months: 97.8% (96.6–98.6%)	
Stage III	3 months: 33.6% (32.7–34.5%)	
	6 months: 88.2% (87.5–88.8%)	
	12 months: 98.9 (98.6–99.1%)	
Primary insurance payor		
Private insurance	3 months: 35.5% (34.4–36.5%)	0.17
	6 months: 89.2% (88.4%–89.9%)	
	12 months: 98.9% (98.5–99.1%)	
Government insurance	3 months: 38.1% (36.7–39.3%)	
	6 months: 87.1% (86.1–88.1%)	
	12 months: 98.7% (98.2–99.1%)	

^a^ 1‐Kaplan–Meier method.

^b^ Logrank test.

## 4 DISCUSSION

To our knowledge, this is the first study evaluating immunotherapy to targeted therapy/chemotherapy in stage III and IV melanoma within the age of modern immunotherapies. We found multiple factors influencing the likelihood and timing of immunotherapy relative to chemotherapy. Multivariable logistic regression analysis revealed that circle distance ≥10 miles, tumor Breslow depth of 1.01–4.0 mm, and disease management in 2016–2018 were factors associated with an increased likelihood of receiving immunotherapy over chemotherapy. Residence in urban areas and stage IV melanoma were associated with a decreased likelihood of receiving immunotherapy. When evaluating time to immunotherapy with multivariable Cox proportional regression analysis, age less than 35, private insurance, and stage IV melanoma were associated with timely receipt of immunotherapy, and minority status and circle distance ≥10 miles were associated with delayed immunotherapy.

In order to capture the association and timing of events, both OR and HR were performed. Patients who travel a greater distance to receive specialized melanoma care (circle distance ≥10 miles) have a higher OR of receiving immunotherapy; however, these patients also experience a delay in immunotherapy receipt compared to those who travel <10 miles (refer to Figures 1 and 2). Prior work has shown that Black Americans showed no difference in their overall odds of receiving immunotherapy for stage IV melanoma relative to NHWs,^5^ but they experienced a delay in immunotherapy initiation.^6^ This study expands these findings to all minority groups and stage III and IV melanoma. These findings are concerning, given the improved overall survival with early/neoadjuvant immunotherapy.^9^

Minority groups have a delay in their initial diagnosis of melanoma and present with more advanced stages.^10^ Blacks have a delay in the surgical treatment of their melanoma compared to NHWs, regardless of insurance status, which may impact overall survival.^6^, ^11^ This report adds to the literature that inequities exist beyond initial melanoma diagnosis and surgical treatment. Future studies examining the impact of delayed immunotherapy on melanoma‐specific survival are warranted.

Our study observed that patients residing in urban areas were less likely to receive immunotherapy for melanoma treatment than those in metro areas. This could be attributed to the complexities of healthcare delivery in urban settings. The NCDB uses the USDA county labels; the USDA defines urban as a population of less than 20,000. Urban areas are characterized by diverse patient populations, more limited healthcare providers, and may have more community practices and fewer academic practices. Together, this may favor conventional chemotherapy. This finding emphasizes the necessity for further research to ensure equitable access to melanoma treatments across all patient populations, regardless of their geographic location.

There were differences between stage III and IV patients that likely contributed to the different rates of immunotherapy. Stage IV patients were less likely to receive immunotherapy, but would receive it quickly if it was prescribed. It has previously been shown in a stage IV‐only cohort that immunotherapy administration is lower for older patients and those with higher Charlson–Deyo comorbidity scores.^5^ Treatment at academic centers and residents within the highest quartile of high school graduation rates had a higher likelihood of receiving immunotherapy.^5^ The stage IV patients in this cohort were older, had higher Charlson–Deyo comorbidity scores, lived in areas with lower high school graduation rates, and were more likely to be treated at community clinics. These factors likely contributed to the differences between these two groups; however, after controlling for these variables in the multivariate analysis, there was still a significant difference, demonstrating that the melanoma stage is an important factor.

Our study has certain limitations, including the exclusion of uninsured patients due to their small sample size. Moreover, while we imply the superiority of immunotherapy over chemotherapy in melanoma treatment, it is important to recognize that the chemotherapy group consists of BRAF/MEK inhibitors, which may be the first‐line treatment in select melanoma cases. The DREAMseq trial did demonstrate that in BRAF‐mutated melanoma, immunotherapy is superior to BRAF/MEK inhibitors^12^; however, there are times when BRAF/MEK are favored. An example could be a patient with a high disease burden who is not expected to survive the lead time needed for immunotherapy to show efficacy. In this case, BRAF/MEK could control initial disease progression with a transition to immunotherapy later. A strength of this study is the comparison of immunotherapy patients to targeted chemotherapy patients instead of comparing them to those without systemic treatment; this removes confounding reasons why patients may not undergo systemic therapy.

## 5 CONCLUSION

Patients who travel ≥10 miles for their melanoma care and minorities have delayed immunotherapy initiation. Future investigations on this topic could help identify what barriers to timely therapeutic delivery exist within these groups and what approaches can be leveraged to narrow these inequities. The initial focus should be minorities and those traveling ≥50 miles for melanoma specialty care, as these groups had the largest equity gap.

## AUTHOR CONTRIBUTIONS

**Gurman S. Dhaliwal:** Conceptualization (equal); data curation (equal); formal analysis (equal); investigation (equal); methodology (equal); project administration (equal); supervision (equal); visualization (equal); writing – original draft (equal); writing – review and editing (equal). **Ahmad B. Shahin:** Project administration (equal); visualization (equal); writing – original draft (equal); writing – review and editing (equal). **Elisabeth S. Lim:** Data curation (equal); formal analysis (equal); methodology (equal). **Lanyu Mi:** Data curation (equal); formal analysis (equal); methodology (equal). **Aaron R. Mangold:** Supervision (equal); visualization (equal); writing – review and editing (equal). **David L. Swanson:** Supervision (equal); visualization (equal); writing – review and editing (equal). **Collin M. Costello:** Conceptualization (equal); investigation (equal); methodology (equal); project administration (equal); validation (equal); visualization (equal); writing – review and editing (equal).

## CONFLICT OF INTEREST STATEMENT

Collin Costello: No relevant disclosures. He has research grant support from Melanoma Research Alliance paid to the institution, current, and DeepX Health paid to the institution, current. David Swanson, MD: Medical Director, DeepX Health. Aaron Mangold, MD: No relevant disclosures. He has consulted for Kyowa, Eli Lilly, Momenta, UCB, and Regeneron in the past, greater than 24 months ago. He has consulted for PHELEC in the past, greater than 12 months. He has consulted for Incyte, Soligenix, Clarivate, and Bristol Myers Squibb in the past, less than 12 months ago. He consults for Argenyx, Boehringer, Janssen, and Ingelheim currently. He consults for Regeneron and Pfizer currently with payments to the institution. He has grant support from Kyowa, Miragen, Regeneron, Corbus, Pfizer, Incyte, Eli Lilly, Aregenx, Palbella, Abbvie, Priovant, Merck in the last 24 months. Beyond 24 months, grant support has come from Sun Pharma, Elorac, Novartis, and Janssen. His current patents include Methods and Materials for assessing and treating cutaneous squamous cell carcinoma (provisional 63–423,254), use of oral JAKi in Lichen Planus (provisional 63/453,065), and Topical Ruxolitinib in Lichen Planus (WO2022072814A1).

## Supporting information


Figure S1:

Table S1:

Table S2:

## Data Availability

All data used in this manuscript is publicly available from the National Cancer Database.
