# Supplementary material for: Factors influencing receipt and time to treatment of immunotherapy relative to chemotherapy in stage III and stage IV melanoma
Source: Cancer Med. 2024 Jan 8;13(1):e6888. doi: 10.1002/cam4.6888 (PMC10807657; doi:10.1002/cam4.6888)
Supplement: Supplementary file 1 — Figure S1: [file CAM4-13-e6888-s002.docx]

| **Supplemental Figure 1: Exclusion Flow Diagram** | |
| --- | --- |
| Positive histology or positive microscopic confirmation  N= 39142  Melanomas diagnosed between 2011-2018  N= 436072  Stage III and IV melanoma cases only  N=57332  Stage III and IV melanoma cases only  N=56489  Positive histology or positive microscopic confirmation  N= 38978  Cases where systemic therapy administered.  N= 15070  Cases where first/only therapy was chemo or immuno  N= 15050   Start with 706158 cases.    Only cases with insurance  N= 14542   \|  \| \| --- \| |  |

Exclude if melanomas not diagnosed between 2011-2019

N= 270086­

Exclude if melanoma cases not in Stage III and IV

N= 378740

Exclude cases where diagnostic confirmation unknown or method other than histologic or microscopic.

N= 843

Exclude if Surgical Procedure at Primary Site other than those specified*

N= 17347

Exclude if histology = 8771: Epitheloid cell nevus

N= 164

Exclude cases where systemic therapy was not administered or if unknown if systemic therapy was administered.

N= 23908

Exclude cases where first or only therapy was could not be determined.

N=20

Exclude uninsured.

N=508

Exclude if race unknown.

N=96

Only cases with insurance

N= 14446

*Codes included for Surgical Procedure at Primary Site
10 Local tumor destruction, NOS
20 Local tumor excision, NOS
  26 Polypectomy
  27 Excisional biopsy
Any combination of 20 or 26–27 WITH
21 Photodynamic therapy (PDT)
  22 Electrocautery
  23 Cryosurgery
  24 Laser ablation
30 Biopsy of primary tumor followed by a gross excision of the lesion (does not have to be done under the same anesthesia)
  31 Shave biopsy followed by a gross excision of the lesion
  32 Punch biopsy followed by a gross excision of the lesion
  33 Incisional biopsy followed by a gross excision of the lesion
  34 Mohs surgery, NOS
  35 Mohs with 1-cm margin or less
  36 Mohs with more than 1-cm margin
45 Wide excision or reexcision of lesion or minor (local) amputation with margins more than 1 cm, NOS. Margins MUST be microscopically negative.
  46 WITH margins more than 1 cm and less than or equal to 2 cm
  47 WITH margins greater than 2 cm
60 Major amputation
90 Surgery, NOS
