# Supplementary material for: Factors influencing receipt and time to treatment of immunotherapy relative to chemotherapy in stage III and stage IV melanoma
Source: Cancer Med. 2024 Jan 8;13(1):e6888. doi: 10.1002/cam4.6888 (PMC10807657; doi:10.1002/cam4.6888)
Supplement: Supplementary file 2 — Table S1: [file CAM4-13-e6888-s001.docx]

Supplemental Table 1: Comparing Chemotherapy and Immunotherapy

|  |  | Chemotherapy (n=2,393) | Immunotherapy (n-12,053) | p-value |
| --- | --- | --- | --- | --- |
| **Group of Age at Diagnosis**, n (%) |  |  |  | 0.18 |
|  | 0-34 years of age | 220 (9.2%) | 1,238 (10.3%) |  |
|  | 35 to 75 years of age | 1,884 (78.7%) | 9,455 (78.4%) |  |
|  | 76 years or older | 289 (12.1%) | 1,360 (11.3%) |  |
| **Sex**, n (%) |  |  |  | 0.40 |
|  | Male | 1,500 (62.7%) | 7,444 (61.8%) |  |
|  | Female | 893 (37.3%) | 4,609 (38.2%) |  |
| **Minority (Y/N)**, n (%) |  |  |  | 0.51 |
|  | No | 2,276 (95.1%) | 11,424 (94.8%) |  |
|  | Yes | 117 (4.9%) | 629 (5.2%) |  |
| **Primary Payor**, n (%) |  |  |  | 0.07 |
|  | Government (Medicaid/Medicare/Other) | 1,027 (43.9%) | 4,980 (41.9%) |  |
|  | Private Insurance | 1,313 (56.1%) | 6,919 (58.1%) |  |
| **Percent No High School Degree Quartiles 2012-2016,** n (%) |  |  |  | <.001 |
|  | Missing | 257 | 1,881 |  |
|  | Greater than or equal to 17.6% | 381 (17.8%) | 1,476 (14.5%) |  |
|  | 10.9-17.5% | 563 (26.4%) | 2,477 (24.4%) |  |
|  | 6.3-10.8% | 666 (31.2%) | 3,255 (32.0%) |  |
|  | <6.3% | 526 (24.6%) | 2,964 (29.1%) |  |
| **Median Income Quartiles, 2012-2016**, n (%) |  |  |  | <.001 |
|  | Missing | 261 | 1896 |  |
|  | Less than $40,227 | 330 (15.5%) | 1,262 (12.4%) |  |
|  | $40,227 – $50,353 | 523 (24.5%) | 2,213 (21.8%) |  |
|  | $50,354 - $63,332 | 517 (24.2%) | 2,611 (25.7%) |  |
|  | Greater than or equal to $63,333 | 762 (35.7%) | 4,071 (40.1%) |  |
| **Population density**, n (%) |  |  |  | 0.008 |
|  | Missing | 60 | 411 |  |
|  | Metropolitan | 1,872 (80.2%) | 9,615 (82.6%) |  |
|  | Rural | 39 (1.7%) | 217 (1.9%) |  |
|  | Urban | 422 (18.1%) | 1,810 (15.5%) |  |
| **Great Circle Distance (categorical)**, n (%) |  |  |  | <.001 |
|  | 10-49 miles | 968 (40.5%) | 4,994 (41.4%) |  |
|  | 50+ miles | 368 (15.4%) | 1,862 (15.4%) |  |
|  | <10 miles | 1,057 (44.2%) | 5,197 (43.1%) |  |
| **Charlson- Deyo Score**, n (%) |  |  |  | 0.58 |
|  | 0 | 1,979 (82.7%) | 9,859 (81.8%) |  |
|  | 1 | 307 (12.8%) | 1,617 (13.4%) |  |
|  | 2 | 72 (3.0%) | 361 (3.0%) |  |
|  | Greater than or equal to 3 | 35 (1.5%) | 216 (1.8%) |  |
| **Year of Diagnosis**, n (%) |  |  |  | <.001 |
|  | 2011 – 2015 | 1,474 (61.6%) | 5,051 (41.9%) |  |
|  | 2016 – 2018 | 919 (38.4%) | 7002 (58.1%) |  |
| **Primary site category**, n (%) |  |  |  | 0.04 |
|  | Missing | 108 | 298 |  |
|  | Head and Neck | 448 (19.6%) | 2,388 (20.3%) |  |
|  | Lower Extremity | 518 (22.7%) | 2,743 (23.3%) |  |
|  | Trunk | 918 (40.2%) | 4,364 (37.1%) |  |
|  | Upper Extremity | 401 (17.5%) | 2,260 (19.2%) |  |
| **Histology category**, n (%) |  |  |  | <.001 |
|  | Acral lentiginous melanoma | 51 (2.1%) | 396 (3.3%) |  |
|  | Blue nevus, malignant | 2 (0.1%) | 3 (0.0%) |  |
|  | Desmoplastic melanoma | 11 (0.5%) | 149 (1.2%) |  |
|  | Lentigo maligna melanoma | 24 (1.0%) | 162 (1.3%) |  |
|  | Magnocellular nevus | 0 (0.0%) | 0 (0.0%) |  |
|  | Mucosal lentiginous melanoma | 0 (0.0%) | 4 (0.0%) |  |
|  | NOS | 1,218 (50.9%) | 4,893 (40.6%) |  |
|  | Nodular melanoma | 603 (25.2%) | 3,354 (27.8%) |  |
|  | Other | 47 (2.0%) | 316 (2.6%) |  |
|  | Superficial spreading melanoma | 437 (18.3%) | 2,776 (23.0%) |  |
| **Breslow’s Depth**, n (%) |  |  |  | <.001 |
|  | Unknown | 395 | 3,194 |  |
|  | 1.01 – 2.0 mm | 322 (16.1%) | 1,858 (21.0%) |  |
|  | 2.01 – 4.0 mm | 410 (20.5%) | 2,399 (27.1%) |  |
|  | Less than or equal to 1.0 mm | 275 (13.8%) | 956 (10.8%) |  |
|  | Greater than 4.0 mm | 991 (49.6%) | 3,646 (41.2%) |  |
| **Systemic, Days from Dx** |  |  |  | <.001 |
|  | N | 2,294 | 11,694 |  |
|  | Median (IQR) | 92.0 (60.0, 132.0) | 101.0 (73.0, 135.0) |  |
|  | Range | 0.0, 1,449.0 | 0.0, 1,571 |  |
| **Days from Diagnosis to the first/only Therapy (Chemo or Immuno)** |  |  |  | <.001 |
|  | N | 2,296 | 11,696 |  |
|  | Median (IQR) | 92.0 (60.0, 132.0) | 102.0 (73.0, 135.0) |  |
|  | Range | 0.0, 1,449.0 | 0.0, 1,571.0 |  |
| **Vital status** |  |  |  | < .001 |
|  | Missing | 372 | 3,128 |  |
|  | Dead | 1,057 (52.3%) | 2,527 (28.3%) |  |
|  | Alive | 964 (47.7) | 6,398 (71.7%) |  |
