# Supplementary material for: Factors influencing receipt and time to treatment of immunotherapy relative to chemotherapy in stage III and stage IV melanoma
Source: Cancer Med. 2024 Jan 8;13(1):e6888. doi: 10.1002/cam4.6888 (PMC10807657; doi:10.1002/cam4.6888)
Supplement: Supplementary file 3 — Table S2: [file CAM4-13-e6888-s003.docx]

Supplemental Table 2: Comparing Stage III and Stage IV Disease

|  |  | **Stage III**  (N=11844) | **Stage IV** (N=2620) | **P-value** |
| --- | --- | --- | --- | --- |
| **Age at Diagnosis**, n (%) |  |  |  | <.001 |
|  | 0 to 34 years of age | 1334 (11.3%) | 126 (4.8%) |  |
|  | 35 to 75 years of age | 9415 (79.5%) | 1938 (74.0%) |  |
|  | 76 years or older | 1095 (9.2%) | 556 (21.2%) |  |
| **Sex**, n (%) |  |  |  | <.001 |
|  | Male | 7174 (60.6%) | 1787 (68.2%) |  |
|  | Female | 4670 (39.4%) | 833 (31.8%) |  |
| **Primary Payor Status**, n (%) |  |  |  | <.001 |
|  | Missing | 163 | 45 |  |
|  | Private Insurance | 7130 (61.0%) | 1115 (43.3%) |  |
|  | Medicaid | 823 (7.0%) | 232 (9.0%) |  |
|  | Medicare | 3510 (30.0%) | 1191 (46.3%) |  |
|  | Other government | 218 (1.9%) | 37 (1.4%) |  |
| **Median Income Quartiles, 2012-2016**, n (%) |  |  |  | 0.001 |
|  | Missing | 1853 | 305 |  |
|  | < $40,227 | 1262 (12.6%) | 332 (14.3%) |  |
|  | $40,227 – 50,353 | 2181 (21.8%) | 559 (24.1%) |  |
|  | $50,354 – 63,332 | 2544 (25.5%) | 586 (25.3%) |  |
|  | ≥$63,333 | 4004 (40.1%) | 838 (36.2%) |  |
| **Percent No High School Degree Quartiles, 2012-2016**, n (%) |  |  |  | 0.011 |
|  | Missing | 1837 | 302 |  |
|  | ≥17.6% | 1469 (14.7%) | 389 (16.8%) |  |
|  | 10.9-17.5% | 2448 (24.5%) | 598 (25.8%) |  |
|  | 6.3-10.8% | 3210 (32.1%) | 717 (30.9%) |  |
|  | <6.3% | 2880 (28.8%) | 614 (26.5%) |  |
| **Charlson-Deyo Score**, n (%) |  |  |  | 0.001 |
|  | 0 | 9763 (82.4%) | 2092 (79.8%) |  |
|  | 1 | 1557 (13.1%) | 367 (14.0%) |  |
|  | 2 | 332 (2.8%) | 102 (3.9%) |  |
|  | ≥3 | 192 (1.6%) | 59 (2.3%) |  |
| **Facility Type**, n (%) |  |  |  | 0.008 |
|  | Missing | 1962 | 205 |  |
|  | Community Cancer Program | 469 (4.7%) | 131 (5.4%) |  |
|  | Comprehensive Community Cancer Program | 2812 (28.5%) | 720 (29.8%) |  |
|  | Academic/Research Program | 4689 (47.4%) | 1166 (48.3%) |  |
|  | Integrated Network Cancer Program | 1912 (19.3%) | 398 (16.5%) |  |
